# Dorsal Intradural Extramedullary Bronchogenic Cysts: A Case Report and Comprehensive Literature Review

**DOI:** 10.7759/cureus.75177

**Published:** 2024-12-05

**Authors:** Julio Escobar-Moreno, Cristopher Ramirez-Loera, Alfonso Durán-Villalobos, Daniel Alberto Reyes Navarro, Armando S Ruiz-Treviño

**Affiliations:** 1 Neurological Surgery, High Specialty Regional Hospital Bajio, León, MEX

**Keywords:** bronchogenic cyst, dorsal intradural cyst, extramedullary cyst, neurospinal tumors, spinal cord lesions

## Abstract

Intradural extramedullary bronchogenic cysts (IEBCs) are exceedingly rare congenital entities, composed of respiratory epithelial cells derived from the anomalous development of the embryonic foregut. Due to their exceptionally low morbidity, only limited cases are available. Consequently, the clinical features and optimal therapeutic approach remain poorly understood.

We report a very unusual case of a 54-year-old Mexican male who initially presented with paraparesis seven years ago, leading to a significant gait disturbance. Conservative therapy was employed, with notable improvements seen after four months of treatment. No additional ambulation assistance was required over the following six years, reflecting a stable progression of the condition. However, the patient experienced a worsening recurrent paraparesis, along with episodic dorsal pain in the last year. Physical examination revealed a reduction in lower extremity strength, with preservation of sensory function. Magnetic resonance imaging (MRI) showed an intraspinal, intradural extramedullary lesion in the dorsal region, extending for at least 50% of the canal. Areas of hyperintensity on the T2 sequence, with a fusion of vertebral bodies at the T3-T4 levels, were observed. A posterior dorsal approach was performed with T2-T4 laminectomy, durotomy, and resection of the cystic lesion, draining the cyst and removing the capsule. Histopathology from the capsule reported a ciliated cyst morphologically characterized by bronchogenic features; immunohistochemistry revealed positivity for cytokeratin cocktail AE1/AE3. Follow-up MRI showed no residual lesion, without further complications.

Surgical resection is the most effective treatment for intraspinal bronchogenic cysts, providing significant symptom relief. Complete removal is ideal, while partial resection may reduce complications in cases with severe adhesions; however, it increases the risk of recurrence. Due to the rarity of the disease, the number of cases is relatively limited. Future studies should strongly consider employing a larger sample size and extending the follow-up period to better understand the spectrum of the disease.

## Introduction

Bronchogenic cysts are rare entities that arise from the congenital remnants of the primitive foregut, resulting from the abnormal separation of the endoderm and notochordal plate during embryogenesis [1]. Several locations are included within their spectrum; however, intraspinal placement is remarkably uncommon [2,3]. The preferred sites of origin for intraspinal cysts depend on the embryological layer responsible for the formation and adhesion of the lesion [4]. Cervical or upper thoracic segments, commonly presenting as intradural extramedullary lesions, have been identified as the preferred anatomical sites [5,6]. The frequent clinical presentation is mainly influenced by neurological impairment, with pain and sensory deficits highlighted upon disposition through the spinal canal [3]. Due to the exceptionally low morbidity of intradural extramedullary bronchogenic cysts (IEBCs), limited reports are available. Consequently, the clinical spectrum and optimal therapeutic approach remain poorly understood, requiring further comprehensive reports.

This report presents a rare case of a 54-year-old male with recurrent paraparesis and episodic dorsal pain, leading to the diagnosis of an intradural extramedullary cyst. Surgical resection via a dorsal approach was performed, with the lesion identified morphologically as having bronchogenic features. Immunohistochemistry revealed positivity for cytokeratin cocktail AE1/AE3. Two-year clinical follow-up demonstrated continued progress, with no additional neurological deficits.

## Case presentation

Patient history

A 54-year-old male with a history of alcohol use and smoking presented to the clinic with a progressively worsening neurological deficit, which had been exacerbated over the past two years. The patient initially experienced weakness in the lower extremities and significant gait disturbance. The diagnostic approach was carried out through neuroimaging studies. Despite identifying a spinal tumor, the patient did not undergo surgical intervention due to personal circumstances. Physical rehabilitation led to partial recovery of ambulation after four months of treatment. However, the neurological deficit gradually worsened with the recurrence of paraparesis, characterized by pronounced lower extremity weakness, gait instability, paresthesia, urinary retention, and constipation.

Neurological examination revealed 2/5 strength in all lower extremity myotomes, accompanied by hypoesthesia extending from the T3 dermatome downward, hyperreflexia, and an extensor plantar response. The upper extremities showed 5/5 strength with normal sensory function.

Neuroimaging and surgical approach

**Figure 1:**
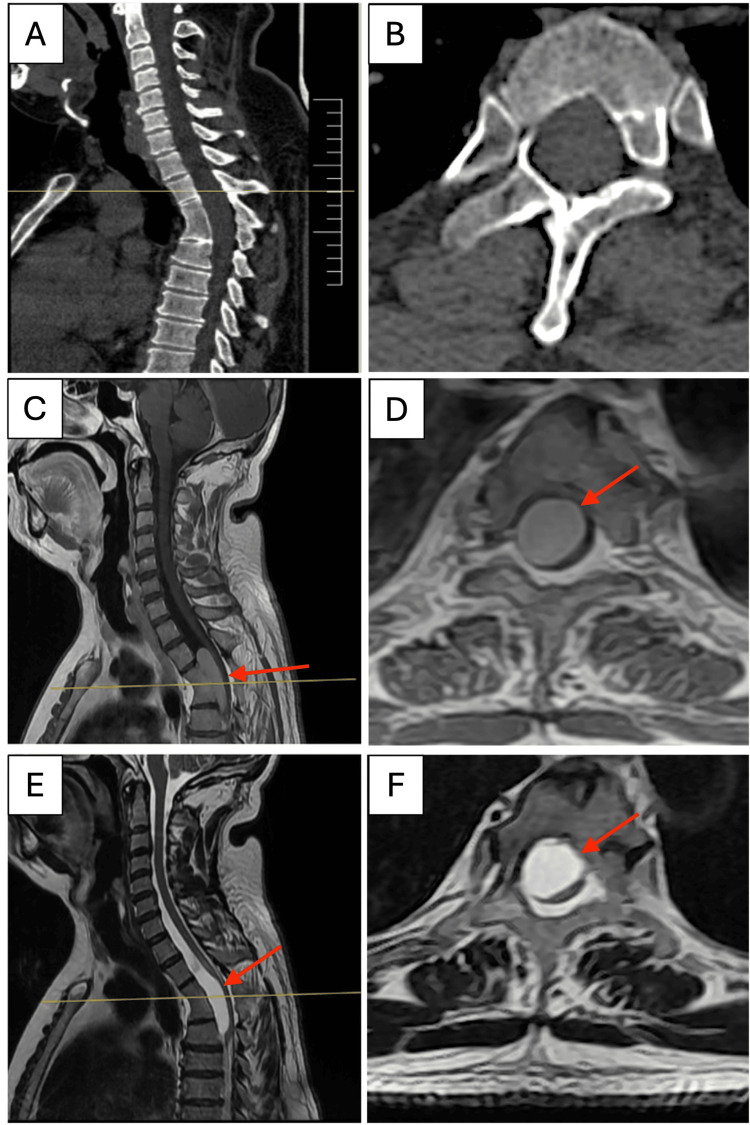
Preoperative imaging (A)-(F) Magnetic resonance imaging (MRI) and computed tomography (CT) scan for diagnostic assessment. (A)-(B) Sagittal and axial CT scan sections show the vertebral fusion at T3-T4 levels, with thoracic kyphosis increased and a widened spinal canal. (C)-(D) MRI at T1 and (E)-(F) T2 sequences with sagittal and axial sections. MRI displayed the fusion of the vertebral bodies at the T3-T4 levels, with a hyperintense lesion within the spinal canal (red arrows). The latter extends from the lower portion of T2-T5 levels, supporting the intradural and extramedullary elements. It also demonstrated the posterior displacement of the spinal cord and an extended area of more than 90% of the spinal canal.

A computed tomography (CT) scan of the spine revealed vertebral fusion at the T3-T4 levels, accompanied by increased thoracic kyphosis and a widened spinal canal, in both sagittal and axial sections (Figure 1).

Magnetic resonance imaging (MRI) at T1-T2 sequences in the sagittal section showed the fusion of the vertebral bodies at T3-T4, with increased thoracic kyphosis and a hyperintense lesion within the spinal canal. The latter extends from the lower portion of the T2-T5 levels, with intradural and extramedullary elements, posterior displacement of the spinal cord, and an extended area occupying more than 90% of the spinal canal.

**Figure 2:**
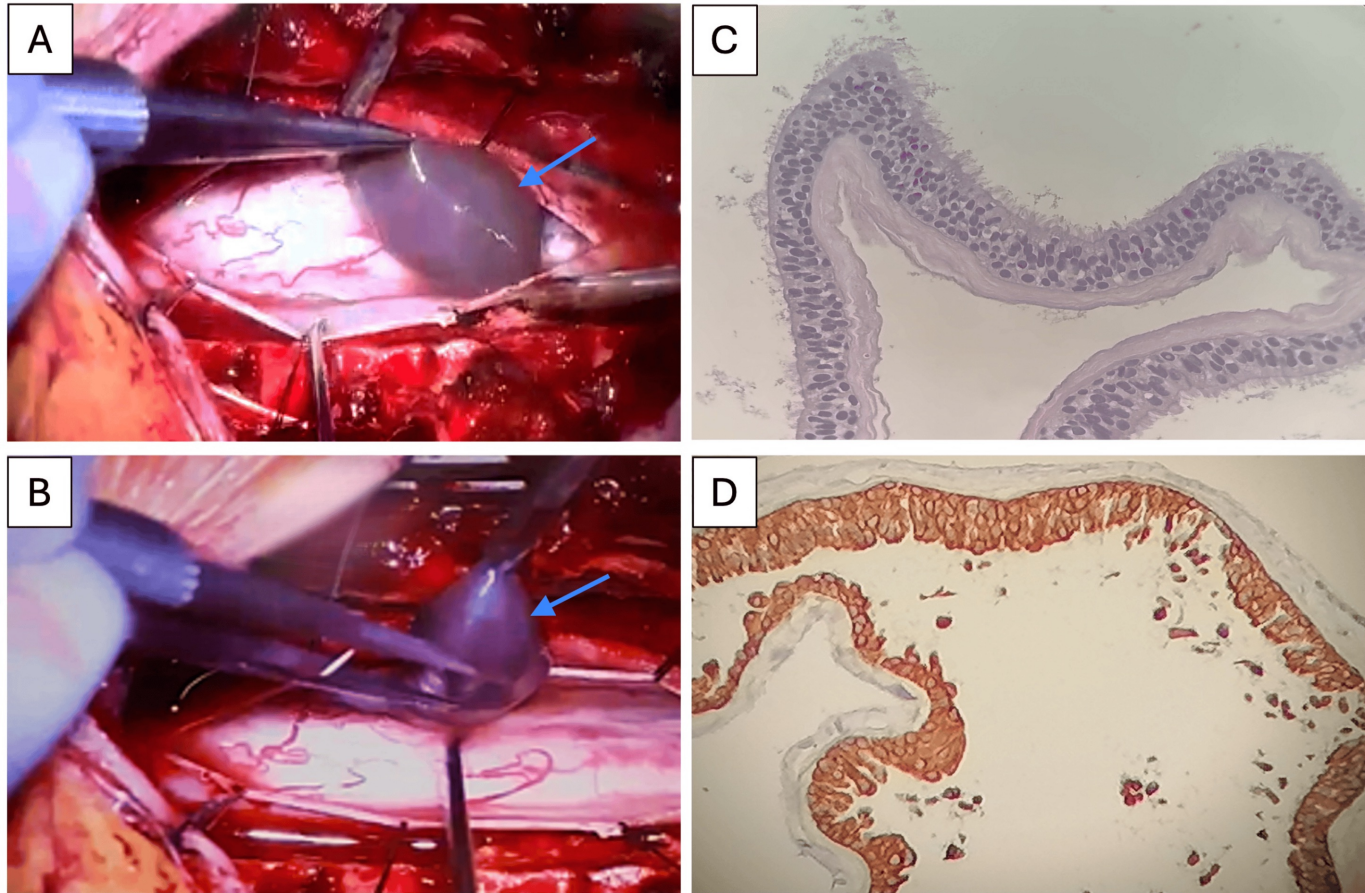
Intraoperative imaging and immunohistopathology visualization (A)-(D) Intraoperative view during surgical resection of the cyst and pathology sections. The intradural extramedullary lesion with a cystic component is visible (blue arrows); complete capsule resection was achieved (A)-(B). Immunohistopathology view (C)-(D). The histopathological study showed a cyst lined by pseudostratified columnar epithelium with cilia on its surface (C). The epithelium rests on the basal membrane (HE stain, 40X). The epithelial lining cells are positive for cytokeratin cocktail AE1/AE3 at 40X magnification (D).

Given the identification of an intradural extramedullary lesion at the T2-T5 levels, the surgical intervention involved a posterior approach, with laminoplasty performed from T2-T5 to ensure adequate exposure of the affected region. After accessing the lesion, meticulous cyst drainage was carried out to decompress the surrounding neural structures and alleviate symptoms. This was followed by a careful capsular resection, with complete removal of the cyst wall to minimize the risk of recurrence while preserving adjacent healthy tissues (Figure 2). Hemostasis was achieved throughout the procedure, and no dural tears were encountered. Postoperatively, the surgical site was closed in anatomical layers, and the patient was monitored for signs of neurological improvement or complications. The surgery was conducted without intraoperative or postoperative complications, with effectiveness and safety in managing the pathology. After the surgical approach, the patient showed significant improvement in strength and sensitivity in both lower extremities.

Postoperative course

The surgical outcome was devoid of complications and resulted in substantial neurological improvement. Motor strength in the lower extremities improved to 4/5 in all myotomes, with sensitivity fully restored. Histopathological examination revealed a bronchogenic cyst with pseudostratified columnar epithelium and cilia; immunohistochemistry was positive for AE1/AE3 cytokeratin, confirming the diagnosis (Figure 2).

After a two-year clinical follow-up, the patient demonstrated continued progress with no additional neurological deficits. This case underscores the significance of considering IEBCs in the differential diagnosis of unexplained neurological deficits, particularly when imaging reveals an intradural lesion. Early surgical intervention, with a cautious suspicion of this entity, can result in significant functional recovery in the long term.

## Discussion

Given the limited number of documented cases, the incidence of bronchogenic cysts, particularly those within the spinal canal, remains underexplored. Liu et al. reviewed available cases, including their institutional experience with 36 patients overall, focusing on evaluating the optimal surgical approach for this entity [3]. Lesions were most commonly located in the cervical and thoracic vertebrae (51.4%), while the lumbar vertebra and thoracolumbar junction accounted for 32.4% and 5.4%, respectively. Axial MRI revealed that the lesions were predominantly dorsal in 19 patients and ventral in 13 patients. All lesions were cystic, without solid components. These observations highlight the critical role of neuroimaging in diagnosis. While the content of the lesions needs to be fully determined, their precise location remains a targeted factor in guiding treatment, with surgical resection being the preferred approach for management. Their findings indicate that the extent of resection does not correlate with improved neurological outcomes; thus, the authors suggest that the degree of resection may not impact symptom remission rates. This report recommends avoiding total resection for lesions with firm adhesions to minimize potential risks. Nevertheless, additional clinical data are needed to establish objective conclusions, especially regarding the appropriateness of different surgical approaches based on anatomical location and lesion origin. Since intramedullary cysts are situated within the spinal cord and extramedullary cysts are located outside the cord, typically within the meninges, accurate neuroimaging is crucial for precise diagnosis and optimal treatment planning [3]. Given the exceptionally low morbidity and the scarcity of clinical reports, our study seeks to address these limitations by providing a detailed review of IEBCs specifically located at thoracic spinal levels.

Since Yamashita et al. first reported the condition in 1973, numerous studies have documented the incidence and demographics of affected populations [7]. Weng et al. presented a series of six patients, pooled with 19 additional cases identified through a comprehensive review [8]. The findings revealed that 56% of IEBCs were diagnosed in individuals during the second and third decades of life, with a male-to-female ratio of 1:1.18. Moreover, 72% and 60% of the cysts were located in the cervical and upper thoracic regions and situated dorsal to the spinal cord, respectively. Surgical findings demonstrated an intramedullary location in 10% of the patients [3,8]. In the present study, 10 cases pooled with one new patient were analyzed, resulting in an 11-patient sample, all with cysts located in the thoracic region; the population consisted only of IEBCs. The median age of diagnosis was 29 years, with 63.7% and 36.3% of patients undergoing gross total resection and partial resection, respectively (Table 1).

**Table 1:** Intradural extramedullary bronchogenic cysts composed of thoracic segments The table presents a detailed compilation and analysis of bronchogenic cysts, with a particular focus on those affecting the thoracic region. The population consisted only of intradural extramedullary bronchogenic cysts. The median age of diagnosis was 29 years, with 63.7% and 36.3% of patients undergoing gross total resection and partial resection, respectively. NR, Not reported; GTR, Gross total resection; PR, Partial resection

Study	Age	Sex	Location	Surgery modality	Follow-up duration (months)
Yamashita et al. [7]	14	Female	C6-CT	GTR	11
Ho and Tiel [9]	21	Female	C5-T3	GTR	NR
Baumann et al. [6]	41	NR	T12-L1	PR	3
Arnold et al. [10]	20	Male	T4	GTR	1
Liu et al. [11]	55	Male	T5-T6	PR	12
Chen et al. [12]	29	Male	T9-T10	PR	NR
Lee et al. [13]	44	Male	T12-L1	GTR	1
Liu et al. [3]	21	Male	T1-T7	PR	23
	10	Male	T2-T3	GTR	60
	55	Male	T2-T3	GTR	3
Current case	54	Male	T2-T5	GTR	24

Neurological deficits appear depending on the location of the cysts. The most common symptom reported with bronchogenic cysts was radiating pain, followed by weakness, with some patients also presenting lower limb atrophy [13-15]. Additionally, previous studies have reported sensory disturbances in the extremities [8], limb weakness, numbness, and paresthesia [11,12,16]. In our study, we focused specifically on lesions that affect the thoracic levels. The present case developed a significant neurological deficit characterized by weakness in lower extremity myotomes, accompanied by hypoesthesia extending from the T3 dermatome downward, hyperreflexia, and an extensor plantar response. This is consistent with the largest pooled patient sample reported in the literature currently [3].

However, clinical diagnosis remains underestimated. The primary diagnostic imaging for bronchogenic cystic lesions includes MRI and CT scans. MRI is best suited for identifying intradural lesions, although there is no consensus on the imaging findings of bronchogenic cysts. This implies the possibility of misdiagnosis and emphasizes the critical need to differentiate these lesions effectively [8]. Spinal arachnoid cysts are predominantly observed in the thoracic region and consistently demonstrate signals comparable to cerebrospinal fluid on MRI [6,8]. Spinal epidermoid cysts are found at the lumbosacral level and exhibit heterogeneous appearances; diffusion-weighted imaging is essential for distinguishing them from other cystic lesions [2]. Mature spinal cystic teratomas, found primarily in children and adolescents, do not possess any distinctive imaging characteristics [8]. Lesions characterized by unclear delineation on imaging were observed in this report, where MRI revealed a hyperintense lesion within the spinal canal, extending from the lower portion of T2-T5 levels. This lesion exhibited distinct intradural and extramedullary elements, although further characterization studies were necessary. Therefore, a definitive diagnosis must be made based on postoperative pathology. In the present case, hematoxylin-eosin staining showed a cyst wall lined by simple and pseudostratified respiratory epithelium, with benign subepithelial mucous glands and fatty components adjacent to the cyst. As immunohistochemistry aids in diagnosing endodermal cysts, the gross sample was positive for AE1/AE3 cytokeratin, confirming the diagnosis [17].

## Conclusions

Surgical resection is the most effective treatment for intraspinal bronchogenic cysts, providing significant symptom relief. Complete removal is ideal, while partial resection may reduce complications in cases with severe adhesions; however, it increases the risk of recurrence. Due to the rarity of the disease, the number of cases is relatively limited. Future studies should strongly consider employing a larger sample size and extending the follow-up period to understand the spectrum of the disease. Study designs that include detailed descriptions of disease progression, recurrence rates, and long-term outcomes are essential for improving diagnostic accuracy and therapeutic strategies.
